# Supplementary material for: Lead-OR: A multimodal platform for deep brain stimulation surgery
Source: eLife. 2022 May 20;11:e72929. doi: 10.7554/eLife.72929 (PMC9177150; doi:10.7554/eLife.72929)
Supplement: Supplementary file 1. [file elife-72929-supp1.pdf]

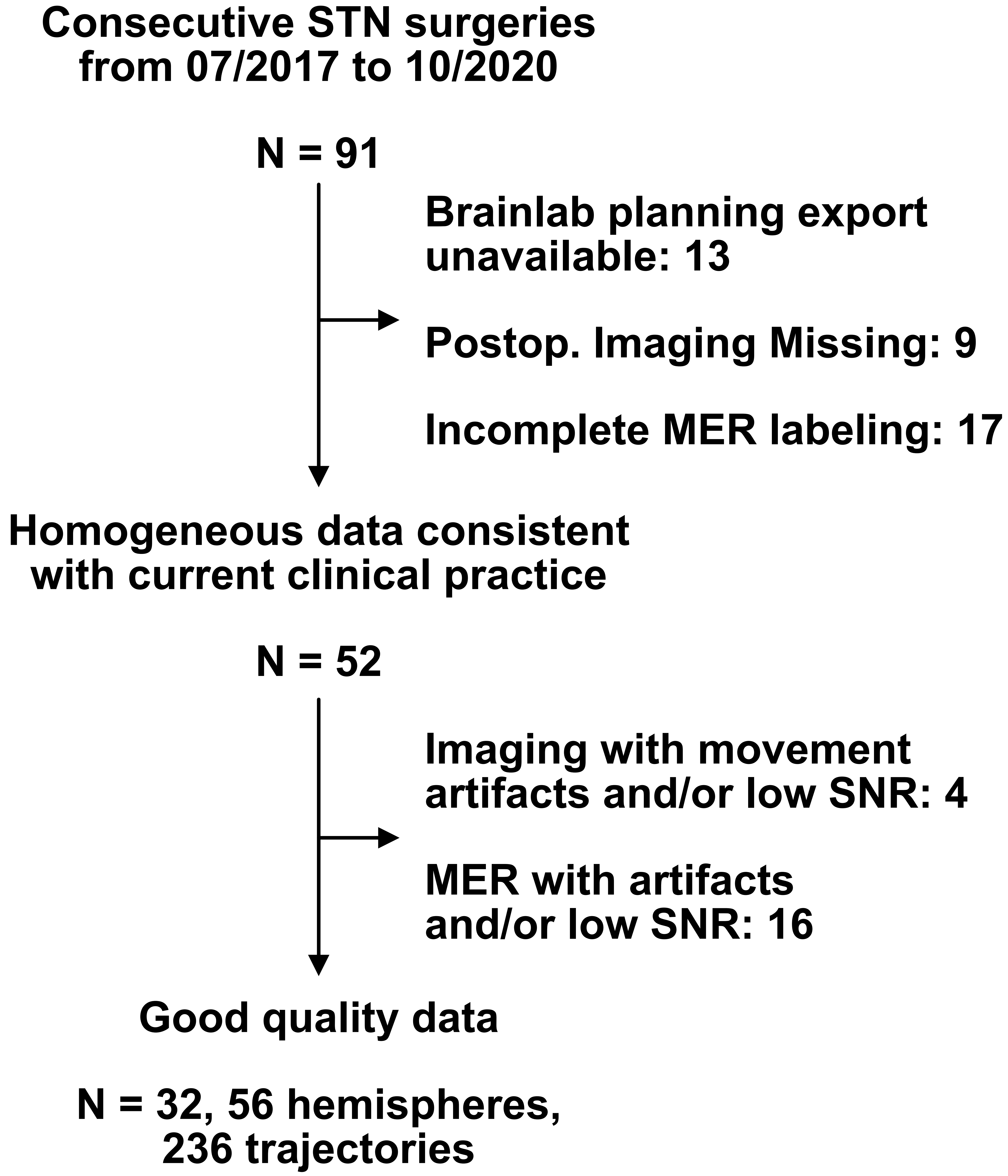
Consecutive STN surgeries
from 07/2017 to 10/2020
N = 91
Brainlab planning export
unavailable: 13
Postop. Imaging Missing: 9
Incomplete MER labeling: 17
Homogeneous data consistent
with current clinical practice
N = 52
Imaging with movement
artifacts and/or low SNR: 4
MER with artifacts
and/or low SNR: 16
Good quality data
N = 32, 56 hemispheres,
236 trajectories
