# Supplementary material for: Lead-OR: A multimodal platform for deep brain stimulation surgery
Source: eLife. 2022 May 20;11:e72929. doi: 10.7554/eLife.72929 (PMC9177150; doi:10.7554/eLife.72929)
Supplement: Transparent reporting form [file elife-72929-transrepform1.pdf]

eLIFE
1st Floor
24 Hills Road
Cambridge CB2 1JP, UK
P 01223 855340
W elifesciences.org
T @elife

eLife's transparent reporting form

We encourage authors to provide detailed information *within their submission* to facilitate the interpretation and replication of experiments. Authors can upload supporting documentation to indicate the use of appropriate reporting guidelines for health-related research (see EQUATOR Network), life science research (see the BioSharing Information Resource), or the ARRIVE guidelines for reporting work involving animal research. Where applicable, authors should refer to any relevant reporting standards documents in this form.

If you have any questions, please consult our Journal Policies and/or contact us: editorial@elifesciences.org.

Sample-size estimation

- You should state whether an appropriate sample size was computed when the study was being designed
- You should state the statistical method of sample size computation and any required assumptions
- If no explicit power analysis was used, you should describe how you decided what sample (replicate) size (number) to use

Please outline where this information can be found within the submission (e.g., sections or figure legends), or explain why this information doesn't apply to your submission:

Since this is a submission within the tools & resources section of eLife, analyses were carried out exploratorily to illustrate potential impact of the novel tool (an intraoperative mapping software). Hence, inclusion number was made based on data availability. Under *Methods, Patient cohort and surgical procedure* (page 7), the patient selection is described.

Replicates

- You should report how often each experiment was performed
- You should include a definition of biological versus technical replication
- The data obtained should be provided and sufficient information should be provided to indicate the number of independent biological and/or technical replicates
- If you encountered any outliers, you should describe how these were handled
- Criteria for exclusion/inclusion of data should be clearly stated
- High-throughput sequence data should be uploaded before submission, with a private link for reviewers provided (these are available from both GEO and ArrayExpress)

Please outline where this information can be found within the submission (e.g., sections or figure legends), or explain why this information doesn't apply to your submission:

Under *Methods, Patient cohort and surgical procedure* (page 7) all of the above is described. In brief, each experiment was only performed once (during clinical routine). Some recordings were discarded due to artifacts in the data (standard procedure for electrophysiological data handling). After that, no outliers were detected, i.e., no specific outlier criteria were applied. High/throughput sequence data does not apply.

eLife Sciences Publications, Ltd is a limited liability non-profit non-stock corporation incorporated in the State of Delaware, USA, with company number 5030732, and is registered in the UK with company number FC030576 and branch number BR015634 at the address 1st Floor, 24 Hills Road, Cambridge CB2 1JP | August 2014

Statistical reporting

- Statistical analysis methods should be described and justified
- Raw data should be presented in figures whenever informative to do so (typically when N per group is less than 10)
- For each experiment, you should identify the statistical tests used, exact values of N, definitions of center, methods of multiple test correction, and dispersion and precision measures (e.g., mean, median, SD, SEM, confidence intervals; and, for the major substantive results, a measure of effect size (e.g., Pearson's r, Cohen's d)
- Report exact p-values wherever possible alongside the summary statistics and 95% confidence intervals. These should be reported for all key questions and not only when the p-value is less than 0.05.

Please outline where this information can be found within the submission (e.g., sections or figure legends), or explain why this information doesn't apply to your submission:

Since this is a submission within the tools & resources section of eLife, focus was on methods development. Hence, few statistical tests were applied. These are reported within legend of Figure 5, as well as results section, on pages 10-11.

(For large datasets, or papers with a very large number of statistical tests, you may upload a single table file with tests, Ns, etc., with reference to sections in the manuscript.)

Group allocation

- Indicate how samples were allocated into experimental groups (in the case of clinical studies, please specify allocation to treatment method); if randomization was used, please also state if restricted randomization was applied
- Indicate if masking was used during group allocation, data collection and/or data analysis

Please outline where this information can be found within the submission (e.g., sections or figure legends), or explain why this information doesn't apply to your submission:

For an illustrative analysis of the electrophysiological pipeline of the tool, continuous NRMS recording data was allocated into the top and bottom 20% to contrast values within the anatomical region of interest (the subthalamic nucleus). This choice was arbitrary and serves to showcase potential applications of the tool to gain major novel biological insights, in the future. Information on this can be found in *Methods, Imaging and electrophysiology processing* (page 8) and the results section (pages 10-11).

Additional data files ("source data")

- We encourage you to upload relevant additional data files, such as numerical data that are represented as a graph in a figure, or as a summary table
- Where provided, these should be in the most useful format, and they can be uploaded as "Source data" files linked to a main figure or table
- Include model definition files including the full list of parameters used
- Include code used for data analysis (e.g., R, Matlab)
- Avoid stating that data files are "available upon request"

Please indicate the figures or tables for which source data files have been provided:

All processed data and code needed to reproduce main findings of the study are made openly available in de-identified form (see figure legends). This can be found in https://github.com/simonoxen/Lead-OR_Supplementary and, when file size allowed, attached to the publication. Due to data privacy regulations of patient data, raw data cannot be publicly shared. Upon reasonable request to the corresponding author, data can be made available after setting up a data sharing agreement between our host institution (Charité — Universitätsmedizin Berlin) and the inquiring party. All code used to analyze the dataset is available within Lead-DBS /-OR software (https://github.com/netstim/leaddbs; https://github.com/netstim/SlicerNetstim)
